# Staging the stands: the ritual choreography of sports fandom and collective emotion regulation

**DOI:** 10.3389/fspor.2026.1805368

**Published:** 2026-04-29

**Authors:** Yuanmei He

**Affiliations:** School of Physical Education, Guangzhou University, Guangzhou, China

**Keywords:** catharsis, choreography, collective behavior, communitas, emotion regulation, mixed-methods, social identity, sports fandom

## Abstract

This study reconceptualizes sports fandom through a performative paradigm, framing avid spectators as co-performers in a collectively choreographed social drama. Using a sequential exploratory mixed-methods design, it first employed field observations and in-depth interviews (*N* = 18) within the highly ritualized fan culture of professional football clubs in South China. Thematic analysis deconstructed the choreographic grammar of fan performances, identifying three core dimensions: Bodily Synchronization, Rhythmic Immersion, and Symbolic Display. These informed the development of a novel 9-item Choreographic Participation Scale (CPS). Following confirmatory factor analysis, a refined 5-item version of the CPS was used in the final structural model, maintaining the three-dimensional structure with improved fit. A subsequent quantitative survey of devoted fans (*N* = 342) integrated the CPS with established measures. Structural Equation Modeling revealed that choreographic participation strongly predicted enhanced social identity (*β* = .66, *p* < .001) and positive affect (*β* = .59, *p* < .001), with significant effects on emotional catharsis and well-being. Mediation analysis confirmed that social identity and positive affect served as significant pathways through which choreographic participation influenced both catharsis and well-being. Crucially, these psychological benefits were robust regardless of match outcome. The findings empirically support Turner's theory of ritual drama, positioning the stadium as a modern liminal space where a “dancing collective body” fulfills fundamental needs for ritualized belonging and emotional synchrony through embodied performance.

## 1 Introduction: from spectacle to sacred performance

The seismic roar of a capacity stadium transcends mere auditory phenomenon; it is the palpable heartbeat of a complex, collective effervescence (1). Within the modern coliseum, a foundational duality unfolds: on the hallowed turf, the Apollonian ethos of order, rational strategy, and measurable outcome reigns supreme (2); in the towering stands, a potent, seemingly anarchic force erupts—a visceral, ecstatic, and profoundly social “Dionysian frenzy.” While the former has been meticulously cataloged, the latter is often reductively framed as primitive tribalism, mass hysteria, or a commodified byproduct of entertainment (3). This framing overlooks the embodied, performative dimensions of fan behavior that have been documented in the growing literature on ultras and supporter cultures (4, 5). This theoretical lacuna obfuscates a deeper truth: to comprehend the enduring power of live sports, we must reconceptualize the fans not as a passive audience, but as primary performers in a vast, improvised ritual dance (6).

We contend that the stadium stands constitute a unique stage, and the spectators, the ardent performers in a collective choreography of belonging. This choreography is composed of synchronized chanting that ebbs and flows like a choral symphony, coordinated kinetic waves that sweep through the terraces, the orchestrated ballet of scarves and flags, and a shared emotional narrative that mirrors—and often intensifies—the dramatic arc of the contest. Its essence is not distant, aesthetic contemplation but deeply embodied co-participation and kinesthetic solidarity (7). This perspective aligns with recent scholarship on football fandom that emphasizes the physical, embodied nature of supporter identity (4, 5).

### 1.1 Theoretical foundations

This perspective necessitates an interdisciplinary synthesis, weaving together anthropological theories of ritual (1, 8), performance studies (6), and the social psychology of groups (9). Specifically, Victor Turner's model of “social drama” (10) provides a masterful framework: the sporting event itself presents the dramatic breach of routine and the mounting crisis of competition, while the fans' synchronized, ritualized responses in the stands constitute the vital redressive action. This collective performance transforms the stadium into a potent liminal space—a threshold realm where mundane social hierarchies and individual identities are temporarily suspended, giving way to *communitas*, a direct, unmediated, and powerfully egalitarian experience of shared humanity (8).

The central psychosocial function of this collective dance, we propose, is emotional integration. It offers a culturally sanctioned, communal vessel for experiencing, expressing, and ultimately metabolizing high-intensity emotions. This aligns with, yet materially expands, the Aristotelian concept of *catharsis* (11) —the purgation of pity and fear through dramatic narrative. In the stadium, catharsis is not achieved through passive observation, but through active, physical immersion in a real, shared, and emotionally charged ritual performance. The rhythmic, repetitive, and synchronous nature of fan behavior reveals profound structural homologies with ritual dances across cultures (12), which are universally recognized for their capacity to induce altered states of consciousness, forge indelible group bonds (1, 13), and mediate collective emotional trajectories (14, 15).

### 1.2 Literature gap and research questions

Despite these rich theoretical consonances, a significant empirical chasm persists. Extant research has described spectator identities (16), analyzed the stadium atmosphere (17), or examined fan engagement in specific cultural contexts (18, 19). However, the specific choreographic dimensions of fan performance—bodily synchronization, rhythmic immersion, and symbolic display—have not been systematically operationalized or linked to emotional outcomes in previous research. To bridge this gap, the present investigation employs a rigorous sequential exploratory mixed-methods design (20).

## 2 Theoretical foundations: ritual, dance, and the collective body

### 2.1 Sport fandom as ritual: empirical foundations

The conceptualization of sport as ritual is a well-trodden path in sociology (16). Drawing on Collins' theory of Interaction Ritual Chains (7), we posit that the stadium functions as a high-density zone of emotional energy. As Turner observed in his analysis of social drama (8), these moments create *communitas*—an unstructured state where social hierarchies dissolve. Beyond these foundational theories, empirical research in sport marketing and sociology has documented specific ritualistic dimensions of football fandom. Dionisio et al. (21) identified collective rituals as central to fan identity construction, while Derbaix and Decrop (22) demonstrated how pre-match, during-match, and post-match rituals serve distinct emotional functions. Tsiotsou (23) further showed that ritual participation predicts team identification and loyalty beyond mere attendance. These studies provide empirical grounding for understanding how the choreographed behaviors observed in the present study function as identity-reinforcing rituals. Recent empirical evidence supports this view, confirming that such costly commitment signals are essential for the stabilization of large-scale groups (15).

### 2.2 Ultras culture and performative fandom

The performative dimensions of fan behavior are most vividly exemplified in ultras culture. Doidge et al. (4) define ultras as organized, dedicated fan groups characterized by coordinated visual and vocal performances, strong group identity, and often political affiliations. Kassing and mean (5) analyze how ultras' choreographed displays—tifos, coordinated chanting, flag displays—function as “identity performances” that simultaneously express and construct collective belonging. Numerato (24) similarly documents how the embodied practices of ultras create boundaries between in-group and out-group, reinforcing solidarity through shared physical expression. This literature provides crucial empirical grounding for the present study's focus on choreographed participation, as the behaviors observed in the stands—precisely timed chants, coordinated visual displays, unified jumping—are the very practices that ultras scholarship has documented.

### 2.3 The kinesthetic collective: dance as muscular bonding

Beyond the psychological, there is a physical dimension. McNeill (12), in his seminal work *Keeping Together in Time*, posited “muscular bonding” —the visceral feeling of solidarity arising from moving in rhythmic synchrony with others—as a fundamental biological mechanism for community formation. This concept has been productively applied in sport fan research; for instance, Derbaix and Decrop (22) describe how shared bodily movements during matches create what they term “collective effervescence,” directly linking physical co-presence to emotional intensity. Recent psychological research supports this, showing that synchrony significantly enhances social connection (13). This aligns with performance studies perspectives (6), suggesting that the “social choreography” (25) of the stands is a deliberate act of nonverbal communication as articulated by Hanna (26).

### 2.4 Embodied cognition and emotional contagion

Theoretical support also comes from embodied cognition and neuroscience. The concept of “kinesthetic empathy” (27) suggests we understand and internalize the movements and emotions of others by unconsciously simulating them. Furthermore, research on emotional contagion (28) and behavioral synchrony (29–31) demonstrates that moving in time with others enhances emotional convergence and social bonding. Tsiotsou (23) integrates these insights in a sport context, showing that the embodied, ritualistic nature of fan participation generates emotional responses that strengthen team identification—a finding that directly informs the present study's hypotheses about the pathways from choreographic participation to social identity and emotional outcomes.

### 2.5 Synthesis and research hypotheses

While existing literature robustly establishes sport as a ritual context and documents the performative practices of organized fan groups, a critical synthesis is lacking. Missing is a unifying framework that treats the core of fan experience as a performative practice with measurable dimensions. The concept of “choreographic participation” integrates insights from ritual theory (Turner, Collins), performance studies (Schechner), and ultras literature (4, 5, 24) to capture the embodied, collective, and symbolic dimensions of fan behavior.

Based on this integrated framework, and consistent with the pathways identified in previous research (22, 23), we hypothesize that:

**H1:** An individual's level of Choreographic Participation will be positively associated with (a) Strength of Social Identity with the fan in-group, (b) intensity of Positive Affect experienced during the match, (c) feelings of Catharsis post-match, and (d) enhanced Subjective Well-being following the event.

**H2:** Social Identity and Positive Affect will serve as significant mediating variables in the relationships between Choreographic Participation and both Catharsis and Subjective Well-being.

**H3:** The positive effects of Choreographic Participation on Catharsis and Subjective Well-being will remain statistically significant even after controlling for the powerful influence of match outcome (win/loss).

## 3 Methodology: a sequential exploratory mixed-methods design

### 3.1 Study context and ethical considerations

This study focuses on the Dragon United FC in the Chinese Super League (CSL). As noted by Giulianotti (16), football spectatorship typologies are complex. The Chinese context offers a distinctive lens on fan engagement. Unlike Western European fan cultures with long-established ultras traditions, Chinese fandom has developed rapidly since the professionalization of the CSL in 1994, blending global fan practices with local cultural emphases on collectivism and emotional restraint. This context potentially amplifies the significance of choreographed participation as a sanctioned outlet for collective emotional expression—a dynamic that is crucial for interpreting the findings of this study. This study was approved by the Institutional Review Board of The University. Written informed consent was obtained from all participants.

### 3.2 Research design

To honor the complexity of the phenomenon—both its rich, subjective texture and its generalizable patterns—we employed a sequential exploratory mixed-methods design (20), combining qualitative observation with quantitative validation. This approach allowed for deep, contextual understanding in Phase 1 to directly inform the instrument development and hypothesis testing in Phase 2.

### 3.3 Phase 1: qualitative exploration — mapping the choreographic grammar

#### 3.3.1 Objective

To develop a rich, emic, and grounded understanding of the choreographic elements, structures, and subjective meanings embedded in fan frenzy. To achieve this, we conducted in-depth interviews and systematic field observations during matches.

#### 3.3.2 Setting and sampling

The research was anchored in the home matches of “Dragon United FC” (a pseudonym), a historic club in the Chinese Super League renowned for its organized, vocal, and visually spectacular fan culture—a prime example of what could be termed “performative fandom.” Over the course of one season, five home matches (representing a mix of wins, draws, and losses) were selected for intensive study. Purposive sampling was employed to achieve theoretical saturation across fan types (32). The sample of 18 participants was strategically constructed to include: recognized “capos” (*n* = 2, to capture leadership perspectives), long-term season-ticket holders in the core active support section (*n* = 12, 5–30 years tenure, to access depth of experience), and newer fans (*n* = 4, <3 years tenure, to capture contemporary entry experiences). Female participants (*n* = 3) were included to address potential gender dimensions of the fan experience. Saturation was assessed through ongoing analysis; by the 15th interview, no new themes emerged, and the final three interviews confirmed thematic completeness.

### 3.4 Data collection

#### 3.4.1 Field observations

The first author systematically observed fan behavior in the core fan section for all five matches. Observation followed a structured protocol (see Supplementary Materials) focusing on: (1) temporal patterns of chanting and movement, (2) spatial propagation of collective actions, and (3) symbolic displays including tifo, flags, and scarves. Data collection involved:

- ***Detailed Field Notes:*** Focused on mapping the kinetic repertoire (e.g., specific jumping patterns, coordinated scarf-waving sequences, the mechanics of the “wave”), the rhythmic and sonic structure (initiation, tempo, and dissolution of chants; call-and-response patterns), and the symbolic deployment (unfurling of TIFOs, use of colored cards, ritualized display of flags at specific moments).
- ***Audio-Visual Recording:*** With prior consent from fan group leaders and in accordance with ethical guidelines, non-intrusive audio and video recordings were made to capture the temporal flow and spatial organization of fan actions for later micro-analysis (see Supplementary Figure S1 for a spatial representation of coordinated fan activities in the North Stand).

#### 3.4.2 Semi-Structured in-depth interviews

Conducted 24–48 h post-match in quiet settings. Interviews, lasting 45–90 min, were designed to elicit thick description of the embodied and emotional experience. Prompts included: “Walk me through your physical actions from entering the stand to the final whistle,” “Describe a moment when you felt most connected to the crowd—what were you doing?” and “How did your body feel after the match, and how did that relate to your emotions?”.

#### 3.4.3 Data analysis

All interview transcripts and field notes were imported into NVivo 12 for analysis. We employed a rigorous Thematic Analysis (33), following a six-phase process: familiarization, initial code generation, searching for themes, reviewing themes, defining/naming themes, and producing the report. Coding was both inductive (open to emergent ideas) and theoretically informed by our framework. Through iterative analysis, the core, overarching theme of “Choreographic Participation” crystallized, organized around three interrelated sub-themes: *Bodily Synchronization* (the conscious and unconscious alignment of one's movements with the surrounding collective), *Rhythmic Immersion* (the subjective experience of being carried by and contributing to the sonic and temporal patterns of the crowd), and *Symbolic Display* (the active, performative use of shared material culture—scarves, colors, gestures—to communicate identity and solidarity).

### 3.5 Phase 2: quantitative validation — modeling the psychosocial pathways

#### 3.5.1 Scale development — the choreographic participation scale (CPS)

The three qualitative sub-themes served as the conceptual blueprint. An initial pool of 12 items was generated (4 items per sub-dimension). While scale development guidelines often recommend larger initial item pools (34), our approach was intentionally focused: items were derived directly from the qualitative findings, with each theme generating items that captured the core meaning expressed by participants. The item development process involved: (1) extracting key phrases from interview transcripts, (2) translating these into declarative statements, (3) reviewing for clarity and representativeness by the research team, and (4) submitting to expert review. The 12-item pool reflects this grounded approach rather than an exploratory over-generation of items. This pool underwent rigorous expert review for content validity by two performance studies scholars and two sport social psychologists. They independently rated each item for relevance, clarity, and representativeness of the theoretical construct. The Item-Level Content Validity Index (I-CVI) exceeded 0.78 for all retained items, indicating strong expert agreement. Items were retained or removed based on expert feedback and cognitive pretesting results. One item was removed for redundancy, one for ambiguous wording, and one for poor fit with the theoretical construct based on multiple expert ratings. The final 9-item scale (3 items per sub-dimension) represents the consensus of expert judgment and participant comprehension. Subsequently, cognitive pretesting was conducted with 10 fans using the “think-aloud” protocol to ensure ecological validity, comprehensibility, and the absence of ambiguous wording. Based on this iterative feedback, the scale was refined to its final 9-item form, measured on a 7-point Likert scale (1 = Strongly Disagree, 7 = Strongly Agree).

#### 3.5.2 Participants and recruitment

The overall research was conducted during the 2024 season. **Phase 1** (qualitative) was carried out from March to May 2024, including field observations at five home matches and in-depth interviews with 18 fans conducted within 48 h post-match.

**Phase 2** (quantitative) was administered during the final months of the regular season, from September to October 2024, capturing fans' evaluations of their season-long experiences. This timing allowed for recruitment at multiple home matches while ensuring that participants could reflect on the full season.

Participants were recruited via two channels:

**Online:** Links to the survey (hosted on Qualtrics) were posted in dedicated Dragon United FC online communities (Weibo supertopic) and general Chinese football forums, with moderator permission. Survey instructions asked participants to reflect on their overall 2024 season experience.

**On-site:** QR codes linking to the same survey were displayed at research booths outside the stadium during three home matches (September 21, October 12, and October 26, 2024), targeting fans pre-match. Survey instructions specified: “Based on your experience attending matches this season.”

Inclusion criteria required participants to be 18+ and self-identify as fans of a professional football team. A small incentive (entry into a lottery for official team merchandise) was offered. Out of 418 initial responses, 342 were retained after rigorous data cleaning: removal of duplicates, straight-lining responses, failures on two embedded attention-check items, and completions in less than one-third of the median time.

The match outcome variable was derived from participants' self-report of the specific match they recalled (for those recruited online and asked about their most recent match) and was included as a control variable in the main structural equation model.

### 3.5.3 Measures

- *Choreographic Participation Scale (CPS):* 9-item, self-developed scale as described. Confirmatory factor analysis was conducted on the full 9-item scale. Based on modification indices and factor loadings, four items were removed in the final structural model due to low factor loadings (<0.50) or cross-loadings > 0.30, resulting in a 5-item scale that maintained the three-dimensional structure with improved model fit (see Results section for details).
- *Social Identity:* 4-item scale adapted from Doosje et al. (35). Sample item: “I identify strongly with other fans of my team” (*α* = .66).
- *Positive and Negative Affect:* We used the 10-item Positive Affect (PA) subscale from the widely validated PANAS (36), with instructions to respond based on feelings “during the match” (*α* = .88). Negative Affect was also measured but was not a central focus in the final model.
- *Catharsis:* A 4-item scale was developed for this study based on theoretical descriptions [e.g., (37),]. Items included: “Watching and participating provided a real emotional release” and “I felt a sense of purification or cleansing after the match” (*α* = .68).
- *Subjective Well-being:* The 5-item Satisfaction with Life Scale [SWLS (38);] was used with a specific timeframe: “Based on how you felt in the hours after the match.” (*α* = .83).
- *Control Variables:* Match outcome (coded: Win = 1, Draw/Loss = 0), fan tenure (in years), age, and gender were included as covariates in the analysis.

### 3.5.4 Analytical strategy

Data were analyzed using SPSS 27 and Mplus 8.3. After descriptive analyses and reliability checks, we performed Confirmatory Factor Analysis (CFA) on the CPS to validate its three-factor structure. Based on CFA results, the CPS was refined to 5 items for the main analyses (see Results). Subsequently, the hypothesized structural model was tested using SEM with maximum likelihood estimation. Model fit was assessed using *χ²*/df ratio, CFI, TLI, RMSEA, and SRMR. To test mediation (H2), bias-corrected bootstrapping with 5,000 resamples was employed. Mediation analysis tested both Social Identity and Positive Affect as parallel mediators, as specified in the revised H2. To test the resilience of effects (H3), match outcome was included as a control variable and as a moderator (CPS  × Match Outcome).

### 3.6 Data integration philosophy

Integration was central to our design. It occurred at two key points: (1) *Building:* Qualitative findings provided the essential, grounded definition and dimensions of the core construct, which was then “built” into the quantitative CPS instrument. (2) *Interpreting:* The quantitative results, particularly the path coefficients and model relationships, are interpreted and given richer meaning through the narrative insights, thick descriptions, and direct quotes from the qualitative phase, allowing for a fuller explanation of the “why” behind the “what.”

The full item list for the Choreographic Participation Scale (CPS), the complete correlation matrix for all study variables, and the qualitative coding framework with supporting data excerpts are provided in the Supplementary Materials (Figure S1, Tables S1, and Supplementary file S1).

## 4 Results

### 4.1 Phase 1 findings: deconstructing the stand's choreography

The qualitative analysis revealed a sophisticated, dynamic performance system. The fan section operated not as a chaotic mob but as a skilled, responsive corps de ballet, with its own directors (capos), repertoire, and narrative arc.

*The Performance Structure:* A typical match revealed a clear dramatic choreography:

- *Prelude (Entry & Anthem):* A gathering rhythm. Fans entered, greeting friends with ritualized handshakes. The club anthem was sung not as a passive listening but as a solemn, swaying collective oath, scarves held aloft—a unifying kinetic and sonic gesture.
- *Development (Open Play):* Characterized by call-and-response. The capo, facing the crowd, initiated chants with specific, exaggerated arm movements that were instantly mirrored by the front rows and rippled backward. Chants followed strict, often complex, rhythmic loops, their tempo increasing during attacking phases. This created a powerful visual and auditory echo, a literal embodiment of alignment.
- *Climax (Goal):* The structured choreography momentarily shattered into what one capo called “beautiful chaos”—an explosive, asynchronous eruption of primal jumping, hugging, and screaming. This was swiftly followed by a ritualized, standardized celebration: a specific victory song, sung in perfect unison while jumping in precise time, restoring synchronous order and formally marking the triumphant moment.
- *Resolution (Post-match):* The finale varied by result. A win prompted a slow, triumphant, marching chant as fans departed. A loss often saw a defiant, slower, yet still unified song—a performative display of resilience and enduring loyalty, transforming grief into collective solidarity.

#### 4.1.1 The subjective experience

Interviews consistently used embodied, transcendent language. A long-term fan (Male, 32) articulated: “When the wave comes, you don't think. Your body just knows. You become a cell in a larger organism. All the petty worries from work, the stress, it all gets physically shaken out in that jumping. The sound swallows you. After a loss, singing together hurts, but it's a clean hurt. It's our hurt.” Another participant described the rhythmic dimension: “DRAG-ON UNI-TED” [“Breathing synchronized with the chants, DRAG-ON (inhale) UNI-TED (exhale)”] (P05). A third reflected on symbolic objects: “My scarf is soaked with the sweat of the 2016 relegation battle” (P11). This quote encapsulates the core mechanisms: loss of self-awareness in synchronous action (leading to social identity), physical release (catharsis), and the transformation of individual emotion through collective ritual.

### 4.2 Phase 2 findings: quantitative modeling of the pathways

#### 4.2.1 Demographic profile

The final sample consisted of 342 valid responses. As detailed in Table 1, the majority of respondents were male (84.5%), reflecting the gender distribution typical of football fandom in the region. The age distribution was skewed towards younger adults, with 43.0% aged 18–25. Regarding fan tenure, over half (52.6%) had supported the team for 3–10 years.

**Table 1:** Demographic characteristics of the quantitative sample (*N* = 342).

Characteristic	Category	Freq	%
Gender	Male	289	84.5%
	Female	53	15.5%
Age Group	18–25 years	147	43.0%
	26–35 years	115	33.6%
	36–45 years	54	15.8%
	46 + years	26	7.6%
Fan Tenure	<3 years	40	11.7%
	3–10 years	180	52.6%
	>10 years	122	35.7%
Match Outcome	Win	180	52.6%
	Draw/Loss	162	47.4%

#### 4.2.2 Measurement model assessment

Confirmatory Factor Analysis (CFA) was conducted to assess the measurement model. Initial analysis of the full 9-item CPS revealed suboptimal model fit (*χ²*/df = 3.42, CFI = 0.88, RMSEA = 0.09). Examination of modification indices and factor loadings indicated that four items performed poorly: two items cross-loaded on multiple factors (>0.30), and two items had factor loadings below 0.50. These items were removed iteratively, resulting in a final 5-item scale (two items for Bodily Synchronization, two for Rhythmic Immersion, and one for Symbolic Display). The reduced scale demonstrated excellent fit (*χ²*/df = 1.82, CFI = 0.96, RMSEA = 0.05) and maintained theoretical coverage of the three dimensions while achieving statistical robustness. For all other constructs, factor loadings exceeded the 0.50 threshold. Internal consistency was acceptable, with Cronbach's *α* values ranging from 0.66 to 0.88 (e.g., CPS *α* = .81), as shown in Table 2. Composite reliability (CR) and Average Variance Extracted (AVE) values met the recommended thresholds of 0.70 and 0.50, respectively.

**Table 2:** Reliability and validity analysis (condensed).

Construct	No. of Items	Factor Loadings (Range)	Cronbach's *α*	CR	AVE
Choreographic Participation (CPS)	5	0.72–0.78	0.81	0.87	0.57
Social Identity	4	0.68–0.75	0.66	0.80	0.50
Positive Affect	7	0.74–0.78	0.88	0.91	0.58
Catharsis	3	0.76–0.80	0.68	0.82	0.61
Subjective Well-being	5	0.74–0.80	0.83	0.88	0.59

CR, Composite Reliability; AVE, Average Variance Extracted.

#### 4.2.3 Descriptive statistics

Means, standard deviations, and correlations are presented in Table 3. Choreographic participation (CPS) was positively correlated with social identity (*r* = .66, *p* < .001), positive affect (*r* = .59, *p* < .001), and catharsis (*r* = .55, *p* < .001), providing preliminary support for H1.

**Table 3:** Descriptive statistics, reliability coefficients, and intercorrelations for main study variables (*N* = 342).

Variable	M	SD	1	2	3	4	5
1. CPS	4.23	1.08	–				
2. SocialID	5.69	0.84	0.66	–			
3. PosAffect	5.11	1.08	0.59	0.47	–		
4. Catharsis	4.81	1.06	0.55	0.46	0.51	–	
5. WellBeing	4.89	1.09	0.38	0.49	0.35	0.63	–

#### 4.2.4 Hypothesis testing via structural equation modeling

The hypothesized structural model was tested using SEM, controlling for fan tenure and match outcome. The model demonstrated a good fit to the data: *χ²* (12) = 25.34, *p* < .05, CFI = .99, TLI = .97, RMSEA = .06 (90% CI:.03,.09), SRMR = .03.

#### 4.2.4.1 Direct effects (H1 and control variables)

The standardized path coefficients are presented in Table 4. Contrary to common assumptions, match outcome did not significantly predict catharsis (*β* = −.05, *p* = .220) or subjective well-being (*β* = −.06, *p* = .175). This indicates that the ritualistic benefits of fandom may be resilient to the team's performance on the field.

**Table 4:** Standardized direct effects in the structural equation model.

Path	*β* (Std. Estimate)	S.E.	*p*-value
**Direct Effects from CPS**			
CPS → Social Identity	0.66	0.04	<.001
CPS → Positive Affect	0.59	0.04	<.001
CPS → Catharsis	0.30	0.06	<.001
CPS → Subjective Well-being	0.04	0.07	0.554
**Direct Effects from Mediators**			
Social Identity → Catharsis	0.14	0.06	0.016
Social Identity → Subjective Well-being	0.41	0.06	<.001
Positive Affect → Catharsis	0.27	0.05	<.001
Positive Affect → Subjective Well-being	0.13	0.06	0.028
**Control Variable**			
Match Outcome → Catharsis	−0.05	0.04	0.220
Match Outcome → Subjective Well-being	−0.06	0.05	0.175

CPS, Choreographic Participation Scale, S.E., Standard Error; Model controls for fan tenure, age, and gender (paths not shown for clarity).

Partially supporting H1, CPS was a potent predictor of social identity (*β* = .66, *p* < .001) and positive affect (*β* = .59, *p* < .001). Crucially, CPS also had significant direct effects on catharsis (*β* = .30, *p* < .001). However, the direct effect on subjective well-being was not statistically significant (*β* = .04, *p* = .554).

##### 4.2.4.2 Mediation effects (H2)

Bias-corrected bootstrapping analysis (5,000 samples) tested the indirect pathways. The results, summarized in Table 5, confirmed H2. There were significant indirect effects of CPS on Catharsis through both Social Identity [*β* = .09, 95% CI (.01,.17)] and Positive Affect [*β* = .16, 95% CI (.09,.23)]. Similarly, significant indirect effects on Subjective Well-being were found via Social Identity [*β* = .27, 95% CI (.19,.35)] and Positive Affect [*β* = .08, 95% CI (.01,.14)].

**Table 5:** Standardized indirect and total effects of choreographic participation (bootstrapping, 5,000 samples).

Effect Type & Path	*β* (Estimate)	95% CI Lower	95% CI Upper	*p*-value
Indirect Effects on Catharsis				
CPS → Social ID → Catharsis	0.09	0.01	0.17	0.021
CPS → Pos Affect → Catharsis	0.16	0.09	0.23	<.001
Total Indirect Effect on Catharsis	0.25	0.15	0.35	<.001
Indirect Effects on Well-being				
CPS → Social ID → Well-being	0.27	0.19	0.35	<.001
CPS → Pos Affect → Well-being	0.08	0.01	0.14	0.019
Total Indirect Effect on Well-being	0.35	0.25	0.44	<.001
Total Effects (Direct + Indirect)				
CPS → Catharsis (Total Effect)	0.55	0.46	0.63	<.001
CPS → Well-being (Total Effect)	0.39	0.29	0.48	<.001

The total effects of CPS on the outcomes were substantial: CPS on Catharsis (Total Effect *β* = .55, *p* < .001) and CPS on Well-being (Total Effect *β* = .39, *p* < .001).

#### 4.2.4.3 Embody the integrated structure of SEM

To formally test H3, we examined the model while controlling for match outcome. As shown in Table 4, the relationship between CPS and Catharsis remained highly significant (*p* < .001) despite the inclusion of match outcome. Furthermore, the interaction term (CPS  × Match Outcome) was non-significant for both Catharsis (*β* = .05, *p* = .25) and Well-being (*β* = .04, *p* = .31). This confirms that the emotional benefits of choreographic participation are consistent regardless of whether the team wins or loses.

## 5 Discussion

### 5.1 Theorizing the choreographic turn: an integrative “ritual-dance-emotion” framework

Our findings do not merely apply a single theoretical lens but actively construct and validate an integrative “Ritual-Dance-Emotion” (RDE) framework. This framework bridges three levels of analysis that are often treated in isolation: the socio-structural level of collective ritual (6), the inter-bodily level of synchronized movement (13), and the intra-psychic level of emotional transformation (15, 35). The sporting event provides the ritual container and dramatic narrative (Turner's social drama). Within this liminal space, fans are not passive recipients but active co-creators of *communitas* through the medium of choreographed participation. This participation—operationalized as Bodily Synchronization, Rhythmic Immersion, and Symbolic Display—serves as the crucial mediating mechanism. It is through this synchronized, collective “dance” that the abstract potential for *communitas* becomes a tangible, embodied reality (the muscle bonding effect), which in turn catalyzes the psychological processes of social identity fusion and emotional catharsis.

The RDE framework suggests a conceptual pathway: Ritual Structure → Choreographic Participation → Embodied Communitas → Emotional Integration. While our cross-sectional data cannot establish causality, the pattern of relationships observed—particularly the robust links between CPS and emotional outcomes, and the significant mediating roles of both social identity and positive affect—is consistent with this theoretical sequence.

### 5.2 The psychophysiology of collective movement: pathways to integration

The significant direct path from CPS to Catharsis suggests an additional, more immediate somatic mechanism rooted in the embodied act of release itself. Beyond the cognitive-affective rewards of belonging, the intense, synchronized physical exertion—the collective jumping, shouting, and rhythmic motion within the sanctioned ritual frame—may directly regulate autonomic nervous system arousal and discharge accumulated psychophysiological tension. This is not merely a psychological byproduct but a distinct neurophysiological pathway to emotional purification.

Emerging research in interpersonal neuroscience substantiates this interpretation. Investigations into behavioral synchrony demonstrate that moving in time with others catalyzes the release of neurochemicals such as endorphins, which are intimately associated with social bonding, analgesia, and the modulation of stress responses (31). The consequent elevation in pain threshold presents a compelling physiological analog to the subjective experience of cathartic release—a literal “shaking off” of somatic and emotional strain. Concurrently, studies on neural synchrony reveal that participation in collective rituals can induce alignment in participants' brain activity, particularly within the salience network and regions governing empathy, reward processing, and interoceptive awareness (39).

The stadium environment, characterized by its overwhelming, rhythmic multi-sensory barrage, creates an optimal ecology for such neurobiological entrainment. This convergence suggests that choreographic participation does more than foster a subjective feeling of unity; it actively induces a transient, shared psychobiological state. The body in synchrony is not just expressing solidarity; it is undergoing a bio cognitive reset.

**The ritual frame: Beyond winning and losing** Perhaps the most socially significant finding is the resilience of these effects to competitive outcome. Results showed that match outcome had no significant impact on the cathartic experience, highlighting that the benefits of choreographic participation are substantial and independent of victory or defeat. This transforms our understanding of fan loyalty. It is not solely a calculation of vicarious achievement (BIRGing) (40), but an investment in a reliable ritual process that provides emotional sustenance regardless of external results. The stadium, in this light, becomes a “container for collective grief” as much as a venue for celebration. The ritualized, shared expression of disappointment performs a crucial integrative function, transmuting private, potentially isolating sorrow into a shared, meaningful, and solidarity-affirming experience (41). This is a contemporary, secular enactment of the tragic catharsis that Aristotle located in the theater, now lived and felt in the collective body of the crowd.

**Implications and future directions** Our findings strongly support Social Identity Theory (9). Consistent with Doosje et al. (35), high identifiers showed greater group cohesion. More importantly, this study contributes to the “Social Cure” perspective (42), demonstrating how group dynamics can mitigate psychological distress (43). The positive correlation between participation and well-being (44) aligns with the growing body of literature on the mental health benefits of sport fandom (45).

**Practical implications** exist for multiple stakeholders. For clubs and leagues, it underscores that the fan culture's organic choreography is not peripheral “atmosphere” but the core emotional engine of the live event (16). Supporting fan groups and respecting choreographic traditions are not just good PR but essential to product integrity. For public health, it identifies organized fandom as a potential source of collective resilience, offering a model for sanctioned, non-verbal communal emotional expression.

**Limitations and future research** Our sample, though sizeable, was culturally specific. This study is grounded in the specific cultural context of Chinese football fandom, which may limit transferability to fan cultures with different historical trajectories and cultural norms. The collectivist orientation of Chinese society may amplify the social bonding effects of choreographed participation relative to more individualistic contexts. Future research should examine these dynamics cross-culturally. While we focused on self-report, we acknowledge the limits of fandom described by Guschwan (46). However, a key finding remains: the benefits of participation persist regardless of match outcome, providing a buffer against the stress of a loss (47, 48). Future studies should incorporate psychophysiological measures (e.g., heart-rate synchrony across fans, cortisol levels) to objectively capture the embodied “entrainment” effect.

## 6 Conclusion

This investigation has demonstrated that the true spectacle of sport often lies not in the contest on the field, but in the elaborate, heartfelt performance unfolding in the stands. The stadium is more than an arena; it is a modern ritual space, a liminal stage where thousands co-author a dynamic choreography of belonging. Through the synchronized language of movement, rhythm, and symbol, fans temporarily transcend their individual selves, merging into a powerful, feeling collective—a communitas. This process is far from trivial; it is a functional, psychobiological mechanism for emotional integration. It amplifies joy, alchemizes sorrow, cements community, and contributes to a sense of well-being. In an era often characterized by digital mediation, social fragmentation, and emotional privatization, the sports stadium endures as one of the last mass-scale spaces dedicated to the physical, collective, and cathartic performance of shared identity. The stands are indeed a stage, and the ritual dance performed upon it remains a vital, pulsating testament to the human need for connection, expression, and collective transcendence.

## Data Availability

The original contributions presented in the study are included in the article/Supplementary Material, further inquiries can be directed to the corresponding author.

## Supplementary material

Supplementary Figure S1 Example of fan choreography during a match.

Supplementary Table S1 Full item list for the Choreographic Participation Scale (CPS) and correlation matrix with all study variables.

Supplementary File S1 Thematic Analysis Coding Framework (complete codebook with definitions and coding rules) and anonymized qualitative data excerpts (extended collection of participant quotes).
