# Supplementary figures and images for: Staging the stands: the ritual choreography of sports fandom and collective emotion regulation
Source: Front Sports Act Living. 2026 Apr 29;8:1805368. doi: 10.3389/fspor.2026.1805368 (PMC13169181; doi:10.3389/fspor.2026.1805368)

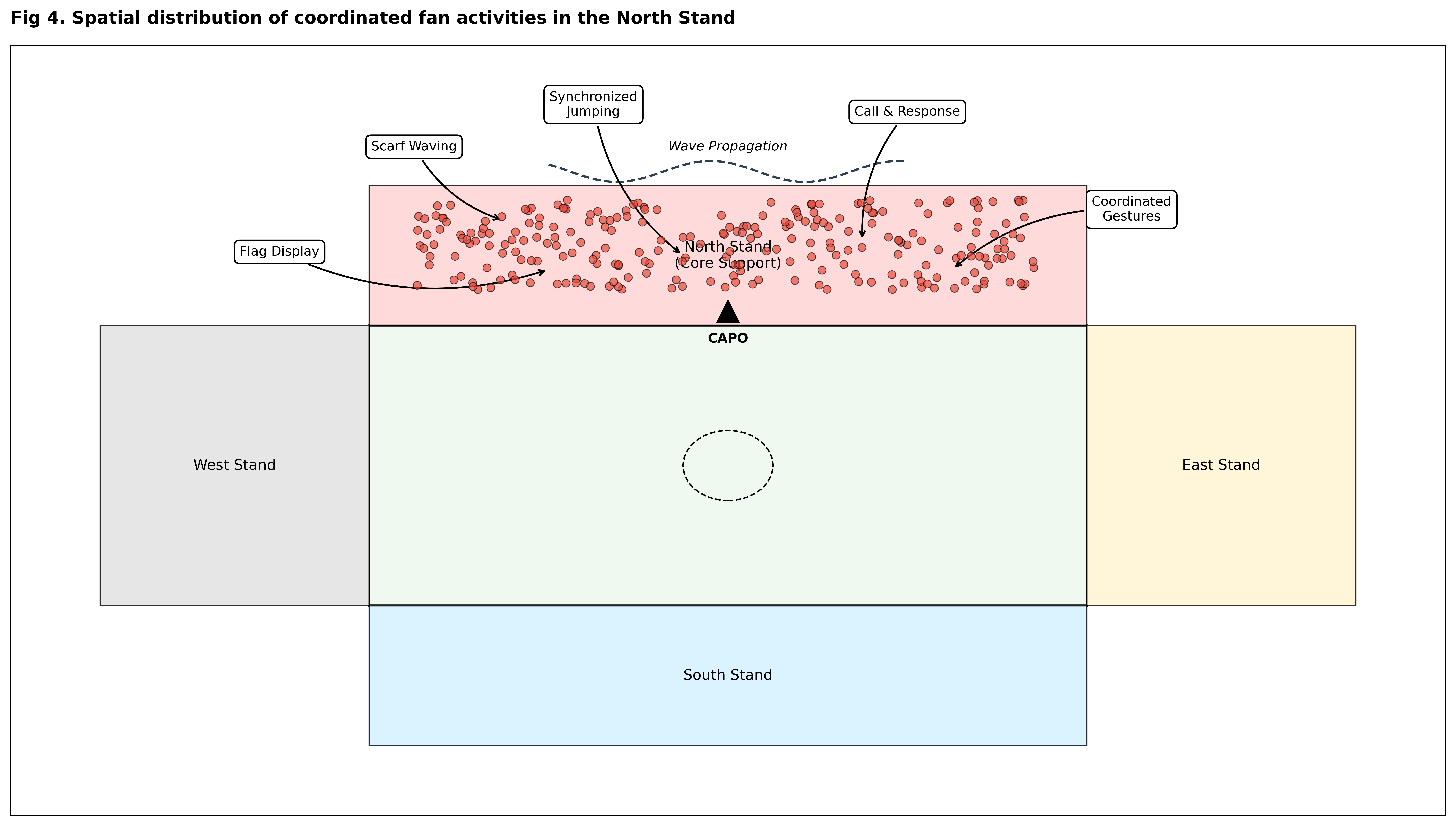

Supplement: Supplementary Figure S1 — Example of fan choreography during a match. [file Image1.png]
