# Supplementary material for: Staging the stands: the ritual choreography of sports fandom and collective emotion regulation
Source: Front Sports Act Living. 2026 Apr 29;8:1805368. doi: 10.3389/fspor.2026.1805368 (PMC13169181; doi:10.3389/fspor.2026.1805368)
Supplement: Supplementary Table S1 — Full item list for the Choreographic Participation Scale (CPS) and correlation matrix with all study variables. [file Table1.docx]

**Table S1. Item list for the 5-item Choreographic Participation Scale (CPS)**

| **Item Code** | **Dimension** | **Item Statement (Survey Original Text)** |
| --- | --- | --- |
| **CPS_1** | Bodily Synchronization | **During the match, I would unconsciously mimic the movements of surrounding fans (e.g., raising arms, jumping).** (Survey Item A1) |
| **CPS_2** | Bodily Synchronization | **I could physically feel the vibration or energy flow of the entire stand.** (Survey Item A3) |
| **CPS_3** | Rhythmic Immersion | **I actively participate in the call-and-response of chants and songs.** (Survey Item B1) |
| **CPS_4** | Rhythmic Immersion | **I feel carried and propelled by the sound of the surrounding crowd, as if floating on a wave of voices.** (Survey Item B3) |
| **CPS_5** | Symbolic Display | **I engage in ritualized use of fan scarves (e.g., unfurling them together before kick-off).** (Survey Item C1) |

**Table S2. Correlation matrix with all study variables**

| **Variable** | **1** | **2** | **3** | **4** | **5** | **6** | **7** | **8** | **9** |
| --- | --- | --- | --- | --- | --- | --- | --- | --- | --- |
| **1. CPS** | – |  |  |  |  |  |  |  |  |
| **2. Social Identity** | .66** | – |  |  |  |  |  |  |  |
| **3. Positive Affect** | .59** | .47** | – |  |  |  |  |  |  |
| **4. Catharsis** | .55** | .46** | .51** | – |  |  |  |  |  |
| **5. Subj. Well-being** | .38** | .49** | .35** | .63** | – |  |  |  |  |
| **6. Match Outcome** (Win=1) | .02 | .03 | .15* | -.05 | -.06 | – |  |  |  |
| **7. Fan Tenure** | .12* | .21** | .08 | .09 | .05 | .01 | – |  |  |
| **8. Age Group** | -.15* | .04 | -.09 | -.06 | -.02 | .00 | .45** | – |  |
| **9. Gender** (Male=1) | .01 | .05 | .02 | .03 | .04 | .02 | .11 | .06 | – |

**A. Thematic Analysis Coding Framework**
*This section illustrates the analytical process from raw data to core themes.*

| **Core Theme** | **Organizing Theme** | **Definition** | **Exemplar Initial Codes** |
| --- | --- | --- | --- |
| **Choreographic Participation** | **1. Bodily Synchronization** | The conscious or unconscious alignment of one's own movements with the rhythm, direction, or form of the surrounding crowd. | “Jumping with the wave”, “mirroring the capo’s gestures”, “not minding shoulder contact with neighbors” |
|  | **2. Rhythmic Immersion** | The subjective experience of being absorbed by and actively contributing to the sonic rhythms (chants, cheers) of the group, leading to a sense of temporal fusion. | “Being swallowed by the singing”, “unconsciously nodding to the beat”, “shouting until hoarse without noticing” |
|  | **3. Symbolic Display** | The active, performative use of shared material culture (scarves, flags, colors) or ritualized gestures to communicate group identity and solidarity. | “Raising scarves in unison at a specific moment”, “wearing identical supporter shirts”, “the collective gesture of pointing to the club crest after a goal” |

**B. Anonymized Data Excerpts and Evidence Chain**
*This section directly links key qualitative claims from the manuscript to de-identified raw data.*

- **Claim 1 (Corresponding to Introduction/Theoretical Foundation):** “The stadium stands constitute a unique stage, and the spectators are the primary performers in a collective choreography of belonging.”
  - **Supporting Excerpt (Field Notes, Match 3):** “Thirty minutes before kick-off, the core stand was nearly full. No one was seated. The crowd was ‘warming up’ in small units—one group rehearsed a new chant rhythm repeatedly; another inspected a large banner (TIFO). It resembled not an audience awaiting a show, but a performing ensemble conducting final rehearsals backstage.”
  - **Analytical Note:** This observation frames the stand as a “backstage/stage” and defines fan activities as “rehearsal,” directly supporting the “performer” metaphor.
- **Claim 2 (Corresponding to the ‘Bodily Synchronization’ theme):** “Synchronous behavior is not entirely spontaneous but is guided and modulated by ‘capos’ (chant leaders).”
  - **Supporting Excerpt (Participant P12, Senior Fan):** “We watch the ‘conductor’ (referring to the capo). He faces us, with his back to the pitch. He raises his arms, and we start chanting; he lowers his hands, and the volume drops; he rotates his arms quickly, and the tempo increases. We are a **human instrument**, and he is the conductor.”
  - **Analytical Note:** This metaphor vividly illustrates the power structure and coordination within the choreography, describing collective action as an organized art form.
- **Claim 3 (Corresponding to the link between ‘Rhythmic Immersion’ and ‘Catharsis’):** “Rhythmic collective action facilitates emotional release and a sense of purification.”
  - **Supporting Excerpt (Participant P05, Female Fan):** “The most exhausted moment is after the final whistle. My voice is hoarse, my arms are sore, but my mind feels peculiarly **clean**, as if all the frustrations from work and the annoyances of daily life have been **shaken out** through the 90 minutes of shouting and jumping. We also cry after a loss, but when everyone is singing through tears, that sadness is different—it is not lonely.”
  - **Analytical Note:** This description directly connects **physical activity** (shouting, jumping), **emotional process** (release, purification), and **social bonding** (not lonely), perfectly embodying the “dance-emotion integration” framework.
