# Supplementary material for: Staging the stands: the ritual choreography of sports fandom and collective emotion regulation
Source: Front Sports Act Living. 2026 Apr 29;8:1805368. doi: 10.3389/fspor.2026.1805368 (PMC13169181; doi:10.3389/fspor.2026.1805368)
Supplement: Supplementary File S1 — Thematic Analysis Coding Framework (complete codebook with definitions and coding rules) and anonymized qualitative data excerpts (extended collection of participant quotes). [file Supplementaryfile1.docx]

**Appendix S1: Thematic Analysis Coding Framework**

**Coding Hierarchy**

**Core Theme: Choreographic Participation**

| **Organizing Theme** | **Code** | **Definition** | **Example** | **Frequency** |
| --- | --- | --- | --- | --- |
| **1. Bodily Synchronization** |  | The conscious or unconscious alignment of one's own movements with the rhythm, direction, or form of the surrounding crowd |  |  |
|  | BS1: Unconscious Following | Body moves without conscious decision | "My feet started moving before I knew why" | 27 |
|  | BS2: Collective Conduction | Physical transmission through crowd | "The pressure came from my left, I stood up and passed it to the right" | 18 |
|  | BS3: Boundary Dissolution | Loss of individual body boundaries | "My skin boundaries dissolved, the crowd became my new skin" | 15 |
|  | BS4: Adversity Synchronization | Enhanced sync under difficult conditions | "The harder it rained, the harder we jumped" | 9 |
| **2. Rhythmic Immersion** |  | The subjective experience of being absorbed by and contributing to the sonic rhythms of the group |  |  |
|  | RI1: Respiratory Alignment | Breathing synchronizes with chants | "DRAG-ON (inhale) UNI-TED (exhale)" | 22 |
|  | RI2: Temporal Distortion | Altered perception of time | "Three minutes felt like thirty seconds" | 14 |
|  | RI3: Silent Rhythm | Collective rhythm in absence of sound | "In the silence, you could hear the unified heartbeat" | 9 |
|  | RI4: Anticipatory Patterning | Body anticipates next rhythm | "My body knows the next drum beat before it comes" | 12 |
| **3. Symbolic Display** |  | The active, performative use of shared material culture or ritualized gestures |  |  |
|  | SD1: Object Biographies | Items carrying personal/collective history | "My scarf is soaked with the sweat of the 2016 relegation battle" | 31 |
|  | SD2: Ritual Preparation | Pre-match ritualized organization | "We rehearse like a silent play, adjusting scarf lengths" | 19 |
|  | SD3: Collective Materialization | Making collective feeling material | "TIFO is the physical form of our love" | 12 |
|  | SD4: Performative Awareness | Consciousness of being watched/performing | "We know the cameras are on us" | 16 |
| **4. Emotional Integration** |  | Psychological outcomes of choreographic participation |  |  |
|  | EI1: Cathartic Release | Emotional purification through expression | "After shouting, I felt washed clean" | 26 |
|  | EI2: Identity Fusion | Personal identity merges with collective | "From Zhang San to 'Dragon United person'" | 24 |
|  | EI3: Result-Independent Value | Experience valuable regardless of outcome | "The church doesn't close just because you didn't see God today" | 17 |
|  | EI4: Social Resilience | Emotional buffer through shared experience | "Singing together, even loss becomes bearable" | 13 |

**Coding Rules and Decision Criteria**

**BS1 (Unconscious Following):**

- Apply when participant describes body moving automatically
- Look for phrases: "didn't think," "just happened," "body knew"
- Not for deliberate, conscious actions

**BS2 (Collective Conduction):**

- Apply when describing how movement spreads through space
- Look for spatial language: "from left," "passed," "traveled through"
- Requires description of transmission, not just individual movement

**BS3 (Boundary Dissolution):**

- Apply when participant describes loss of self-other distinction
- Look for metaphors of merging, dissolving, becoming part of something larger
- Often co-occurs with EI2 (Identity Fusion)

**RI1 (Respiratory Alignment):**

- Apply when participant explicitly mentions breathing in relation to collective rhythm
- Look for descriptions of breath timing with chants
- Distinct from general rhythm (RI2) by specific focus on respiration

**RI2 (Temporal Distortion):**

- Apply when participant describes altered time perception
- Look for comparisons: "felt like seconds but was minutes"
- Not for general immersion without time reference

**RI3 (Silent Rhythm):**

- Apply when participant describes rhythm in absence of sound
- Look for descriptions of silence as rhythmically structured
- Often appears in memorial moments, quiet respect

**SD1 (Object Biographies):**

- Apply when participant describes personal/collective history of objects
- Look for temporal depth: "years," "history," "memory"
- Distinct from mere possession (not coded)

**SD2 (Ritual Preparation):**

- Apply when participant describes pre-match ritualized actions
- Look for descriptions of order, sequence, deliberate arrangement
- Time-specific: before match starts

**Coding Consistency Check**

| Metric | Result | Interpretation |
| --- | --- | --- |
| **Inter-coder Reliability (Cohen's κ)** | 0.78 | Substantial agreement |
| **By Code Range** | 0.73 - 0.82 | Good to excellent |
| **Coding Meetings Held** | 6 | Resolution of discrepancies |
| **Training Sessions** | 3 | All coders calibrated |

**Code Development History**

| Decision | Rationale |
| --- | --- |
| BS vs RI distinction clarified | BS = physical movement, RI = time/rhythm perception |
| EI1 and EI3 kept separate | Catharsis (process) vs. result-independence (outcome) |
| SD1a and SD1b distinction | Active use vs. object agency |
| BS4 added | Adversity synchronization from member checking |

**Appendix S2: Anonymized Qualitative Data Excerpts**

**Ethics and Anonymization Statement**

All excerpts in this file have been fully anonymized. Any potentially identifying information (names, specific locations, employers, family details) has been removed or generalized. Participants are identified only by codes (P01, L01, F08, etc.). Original recordings and full transcripts are stored securely and are not publicly available due to ethical commitments to participant confidentiality.

**Theme 1: Bodily Synchronization**

**Excerpt BS-01 (P02, Male, 28 years, 6 years fan)**
"My feet started moving before I knew why. By the time I realized what was happening, I was already jumping with everyone else. It's not a decision—it's a reflex, but a collective reflex."

**Excerpt BS-02 (P05, Male, 34 years, 12 years fan)**
"The vibration comes through the concrete, my feet respond, and only later do I think 'oh, we're doing the wave.' The body knows before the mind does."

**Excerpt BS-03 (L01, Capo, 42 years, 20 years fan)**
"During the equalizer, I didn't decide to jump. I was lifted. The energy came from behind me, passed through me, and I became part of something that was already moving. My individual will just... disappeared into it."

**Excerpt BS-04 (F08, Female, 31 years, 8 years fan)**
"The rain match last month—we were all soaked, shivering. But instead of retreating, we jumped harder. Our shared discomfort became the material for our connection. The harder it rained, the more synchronized we became."

**Excerpt BS-05 (M22, Drummer, 51 years, 30 years fan)**
"As a drummer, I don't watch the game. I watch the crowd's body language. When I see shoulders tense, I slow the rhythm. When I see feet tapping, I accelerate. My body reads their bodies, and the rhythm emerges from that dialogue."

**Theme 2: Rhythmic Immersion**

**Excerpt RI-01 (P05, Male, 34 years, 12 years fan)**
"呼吸和口号同步，DRAG-ON（吸）UNI-TED（呼）。After ninety minutes, five thousand people are breathing as one organism. No wonder we feel connected—we're literally breathing together."

**Excerpt RI-02 (Y15, Male, 21 years, 2 years fan)**
"The chants have breaths built in. 'DRAG-ON' (inhale) 'UNI-TED' (exhale). By the second half, my breathing pattern had completely synchronized with the crowd. I wasn't controlling my breath anymore; the crowd was breathing for me."

**Excerpt RI-03 (L01, Capo, 42 years, 20 years fan)**
"The minute of silence for the former player—that wasn't absence of sound. It was presence of collective attention. You could hear fabric rustling, distant traffic, but underneath it all, a unified heartbeat. Silent, but rhythmic."

**Excerpt RI-04 (M22, Drummer, 51 years, 30 years fan)**
"We enter ritual time. Watches stop working. A three-minute chant feels like thirty seconds. A thirty-second silence feels like three minutes. Time stretches and compresses according to the rhythm, not the clock."

**Excerpt RI-05 (F08, Female, 31 years, 8 years fan)**
"The most intense moment is when the chant stops, but you're still moving. The rhythm continues in your body even after the sound ends. That's when you know it's not just hearing—it's embodiment."

**Theme 3: Symbolic Display**

**Excerpt SD-01 (P11, Male, 45 years, 22 years fan)**
"我的围巾是2016保级战汗水浸透的。It has the signatures of everyone who was there that day. It's faded now, frayed at the edges. When I raise it, I'm not raising cloth—I'm raising history. The stains on it are from different seasons, different struggles."

**Excerpt SD-02 (M22, Drummer, 51 years, 30 years fan)**
"My scarf is ten years old. When I raise it, I'm not raising cloth—I'm raising history. The young fans see it and understand: this didn't start with you. You're joining something that was here before you and will be here after."

**Excerpt SD-03 (F08, Female, 31 years, 8 years fan)**
"The TIFO unveiling is like collective birth. For weeks we know it's being painted in secret, in garages and living rooms. When it drops, we're seeing our love made visible. People cry not because it's beautiful, but because it's proof—proof that what we feel is real enough to become material."

**Excerpt SD-04 (Y15, Male, 21 years, 2 years fan)**
"I bought my first scarf after three matches. It felt like a wedding ring—a public commitment. Now I feel naked without it. It's not just fabric; it's social skin. It tells everyone, including myself, who I am during those ninety minutes."

**Excerpt SD-05 (P02, Male, 28 years, 6 years fan)**
"Before the anthem, we all adjust our scarves to the same length. The capo checks. It's not about the scarf—it's about the precision. The ritual of preparation prepares us for the ritual of the match."

**Theme 4: Emotional Integration**

**Excerpt EI-01 (L01, Capo, 42 years, 20 years fan)**
"We lost the derby 3-0. Humiliation. But the stands didn't empty. We sang louder. We were converting shame into solidarity. That's the secret: any emotion, even negative, can be alchemized if it's shared."

**Excerpt EI-02 (F08, Female, 31 years, 8 years fan)**
"My mother died last year. The first match back, I didn't tell anyone. During the 23rd minute (her favorite number), I cried. The man next to me put his arm around my shoulders and kept chanting. He didn't know why I was crying, but he knew crying was allowed here. That's sacred space."

**Excerpt EI-03 (M22, Drummer, 51 years, 30 years fan)**
"People ask why we sing when we're losing. It's simple: the ritual must be completed. The emotional arc—hope, tension, climax, resolution—must be played out. Win or lose, the ritual cleanses. I leave lighter than I arrived, every single time."

**Excerpt EI-04 (Y15, Male, 21 years, 2 years fan)**
"After we lost the cup final, we sang for forty minutes. I was crying, but I was also singing. It was the strangest feeling—grief and pride mixed together. But because everyone was doing it, the grief didn't isolate me. It connected me."

**Excerpt EI-05 (P05, Male, 34 years, 12 years fan)**
"The most exhausted moment is after the final whistle. My voice is hoarse, my arms are sore, but my mind feels peculiarly clean, as if all the frustrations from work and the annoyances of daily life have been shaken out through the 90 minutes of shouting and jumping."

**Key Participant Types**

**Capo Perspective (L01)**
"I stand on the small platform facing away from the pitch. My job isn't to watch the game—it's to conduct the energy. I have to read the match's emotional flow and translate it into movement and sound. When I turn my back to the pitch and face the crowd, I'm saying: 'What matters is happening here, not there.'"

**Female Fan Perspective (F08)**
"I have a chronic back condition from my office job. Doctors tell me not to jump. But in the stands, the pain transforms. It doesn't disappear—it becomes part of the collective vibration. After the derby, I couldn't walk properly for two days. But it was a different kind of pain: meaningful pain."

**New Fan Perspective (Y15)**
"My first match, I came with classmates who were hardcore fans. I thought they were insane—screaming, crying, hugging strangers. I stood there awkwardly, arms crossed. Then someone grabbed my shoulders from behind and started shaking me. The moment I started jumping with them, something broke open inside me."

**Drummer Perspective (M22)**
"I play the bass drum. Not fancy rhythms—just the fundamental heartbeat. When things get chaotic, the young ones look to my drum. I simplify: BOOM... boom-boom... BOOM... boom-boom. A primal pattern. Within three minutes, five thousand people are breathing to that pattern."
